# Clinical determinants of the severity of Middle East respiratory syndrome (MERS): a systematic review and meta-analysis

**DOI:** 10.1186/s12889-016-3881-4

**Published:** 2016-11-29

**Authors:** Ryota Matsuyama, Hiroshi Nishiura, Satoshi Kutsuna, Kayoko Hayakawa, Norio Ohmagari

**Affiliations:** 1 Graduate School of Medicine, Hokkaido University, Kita 15 Jo Nishi 7 Chome, Kita-ku, Sapporo, 060-8638 Japan; 2 CREST, Japan Science and Technology Agency, 4-1-8, Honcho, Kawaguchi-shi, Saitama 332-0012 Japan; 3 Disease Control and Prevention Center, National Center for Global Health and Medicine Hospital, 1-21-1 Toyama, Shinjuku-ku Tokyo, 162-8655 Japan

**Keywords:** Case fatality ratio, Middle East respiratory syndrome, Comorbidity, Ascertainment bias

## Abstract

**Background:**

While the risk of severe complications of Middle East respiratory syndrome (MERS) and its determinants have been explored in previous studies, a systematic analysis of published articles with different designs and populations has yet to be conducted. The present study aimed to systematically review the risk of death associated with MERS as well as risk factors for associated complications.

**Methods:**

PubMed and Web of Science databases were searched for clinical and epidemiological studies on confirmed cases of MERS. Eligible articles reported clinical outcomes, especially severe complications or death associated with MERS. Risks of admission to intensive care unit (ICU), mechanical ventilation and death were estimated. Subsequently, potential associations between MERS-associated death and age, sex, underlying medical conditions and study design were explored.

**Results:**

A total of 25 eligible articles were identified. The case fatality risk ranged from 14.5 to 100%, with the pooled estimate at 39.1%. The risks of ICU admission and mechanical ventilation ranged from 44.4 to 100% and from 25.0 to 100%, with pooled estimates at 78.2 and 73.0%, respectively. These risks showed a substantial heterogeneity among the identified studies, and appeared to be the highest in case studies focusing on ICU cases. We identified older age, male sex and underlying medical conditions, including diabetes mellitus, renal disease, respiratory disease, heart disease and hypertension, as clinical predictors of death associated with MERS. In ICU case studies, the expected odds ratios (OR) of death among patients with underlying heart disease or renal disease to patients without such comorbidities were 0.6 (95% Confidence Interval (CI): 0.1, 4.3) and 0.6 (95% CI: 0.0, 2.1), respectively, while the ORs were 3.8 (95% CI: 3.4, 4.2) and 2.4 (95% CI: 2.0, 2.9), respectively, in studies with other types of designs.

**Conclusions:**

The heterogeneity for the risk of death and severe manifestations was substantially high among the studies, and varying study designs was one of the underlying reasons for this heterogeneity. A statistical estimation of the risk of MERS death and identification of risk factors must be conducted, particularly considering the study design and potential biases associated with case detection and diagnosis.

**Electronic supplementary material:**

The online version of this article (doi:10.1186/s12889-016-3881-4) contains supplementary material, which is available to authorized users.

## Background

Cases of Middle East respiratory syndrome (MERS), caused by MERS-associated coronavirus (MERS-CoV), have continuously been reported since June 2012. As of 29 June 2016, the total number of laboratory-confirmed cases notified to the World Health Organization (WHO) reached 1,768 cases, including 630 deaths [1]. Particularly large outbreaks of MERS-CoV infection have been reported in the Kingdom of Saudi Arabia (KSA) and the Republic of Korea (ROK), while smaller outbreaks and importation events have been reported in other 25 countries [1]. Of these, 10 countries are located in the Middle East, 7 countries in Europe, 3 countries in Africa, 3 countries in Southeast and East Asia, and 1 in North America (the United States of America) [2–4]. Because of the regular reporting of MERS cases in the Middle East, countries across the world are now facing a continuous threat of MERS outbreak.

To understand the clinical burden of MERS, it is necessary to quantify the risk of developing severe clinical manifestations. The case fatality risk (CFR) is a measure of the risk of death among those who satisfy the case condition [5], while risks of admission to an intensive care unit (ICU) and that of requiring mechanical ventilation are also useful to measure the extent of developing severe MERS complications. However, it is not only necessary to estimate such risks, but it is also critically important to identify epidemiological determinants of those risks to then predict the risk of severe complications for each patient before the onset of disease exacerbation [6]. In previous studies, the risk of death among secondary cases was estimated based on statistical modelling and was found to range from 20 to 22%, approximately [7–11]. Meanwhile, among the primary cases, the risk of death was estimated to be greater at approximately 40%, perhaps because of biases associated with case detection and diagnosis [6–8]. As for epidemiological determinants of MERS death, elderly patients with underlying comorbidities have been identified as the most susceptible population with a high risk of death [6, 9, 11].

Despite our further understanding of the risk of developing severe MERS, the abovementioned estimates are mostly based on a subset of MERS cases; for instance, some of the risk estimates are a result of the analysis of cases diagnosed in 2015 in the ROK or KSA alone. Published articles with different study designs and populations have yielded different estimates and effect sizes associated with MERS death. Because of this variability, it is valuable to comprehensively and systematically analyze published MERS studies that have recorded the clinical prognoses of cases. A systematic review is a highly informative review method that combines published results from different studies, thereby merging and contrasting results across multiple studies and answering study questions using the pooled estimates [12]. Thus, we aimed to perform a systematic review to assess risks of death and other severe complications and determine the risk factors for MERS-associated death and contrast these results by study population and study design.

## Methods

The present study was a systematic review conducted in accordance with the Preferred Reporting Items for Systematic Reviews and Meta-Analyses (PRISMA) statement [13]. PICO statement: Our study question is focused on laboratory confirmed cases of MERS regardless of their treatment status, and thus, involves only retrospective observational studies, measuring their risks of admission to Intensive Care Unit (ICU) and death and comparing those risks by age, gender and underlying comorbidities.

### Search strategy

Our systematic review protocol is summarized as Additional file 1. Published studies that referred to the clinical prognosis of MERS cases were retrieved from MEDLINE (PubMed) and Web of Science electronic databases on 16 May 2016. The following search terms were used in “All fields” to identify relevant published articles:

1. “MERS” OR “middle east respiratory syndrome” OR “novel coronavirus” OR “novel coronavirus 2012”
2. “sever*”OR “fatal*”OR “death” OR “mortalit*”
3. “hospitalization” OR “intensive care” OR “ICU”
4. 1 AND 2 AND 3

We limited the search to articles published between April 2012 (i.e., after the first MERS case was reported) and June 2016. Additional studies reporting associated outcomes that were not identified by the abovementioned search strategy were manually retrieved by tracking the references of included articles (i.e., ancestry and discordancy approach). We restricted ourselves to publications written in English.

### Study selection

All titles identified by the abovementioned search strategy were independently screened by two authors (RM and HN). Abstracts of potentially relevant articles were subsequently reviewed for eligibility, and if a description of severe or lethal MERS was available, articles were selected for closer examination of the full text. To be eligible for inclusion, published studies were required to meet the following characteristics: (i) studies focused on patients infected with MERS-CoV and (ii) explicitly documenting clinical outcomes (i.e., prognosis) and characteristics of both surviving and deceased patients. Studies that allowed us to stratify the risk of severe or fatal MERS by demographic or medical condition were preferred, but this was not an essential inclusion criterion. To calculate the risk of severe MERS or MERS death, we excluded case reports that documented only one or two cases (i.e., case reports with a sample size n ≥ 3 were eligible).

Included studies were further classified into five groups based on the study design and population studied: (i) case reports comprising published studies that described the clinical course of individual patients including mild cases; (ii) studies including only ICU cases (hereafter referred to as ICU studies): case reports or retrospective studies that reported outcomes of patients admitted to the ICU only; (iii) hospital studies: retrospective or descriptive studies that aimed to document the outbreak in a hospital or healthcare-associated facility; (iv) retrospective studies: published studies that retrospectively analyzed the series of MERS cases that were registered in the patient database or tracked medical records; and (v) surveillance studies: published studies that extracted data from a database of cases, systematically gathering epidemiological data, as coordinated by a country or WHO.

### Data extraction and analysis

The primary data extracted were the proportions of deceased MERS patients, patients admitted to the ICU and patients undergoing mechanical ventilation. All of these outcomes were dealt with as dichotomous variables, and thus, we calculated the 95% confidence interval (CI) for each included study using the binomial distribution. Whenever possible, we stratified the risk of death by age, sex, underlying medical condition and study design. For the analysis of the effect of each covariate on the outcome, the odds ratio (OR) for death among those with underlying conditions was calculated and compared with those without comorbidities. Stratified analysis could not have been made for the proportions of ICU admission and mechanical ventilation because the dataset of such covariates was not commonly available for these two outcomes. We employed a fixed effects inverse variance weighted model. Weighted means (i.e. pooled estimate) of the abovementioned proportions and the OR for death by each covariate were calculated using the inverse of variance estimates from each study. The heterogeneity among identified studies was statistically assessed by the I^2^ statistic. To explore the possible sources of heterogeneity, we stratified pooled estimates by study design. A forest plot was used to illustrate the distribution of the outcome and effect size obtained from each published study.

## Results

**Fig. 1:**
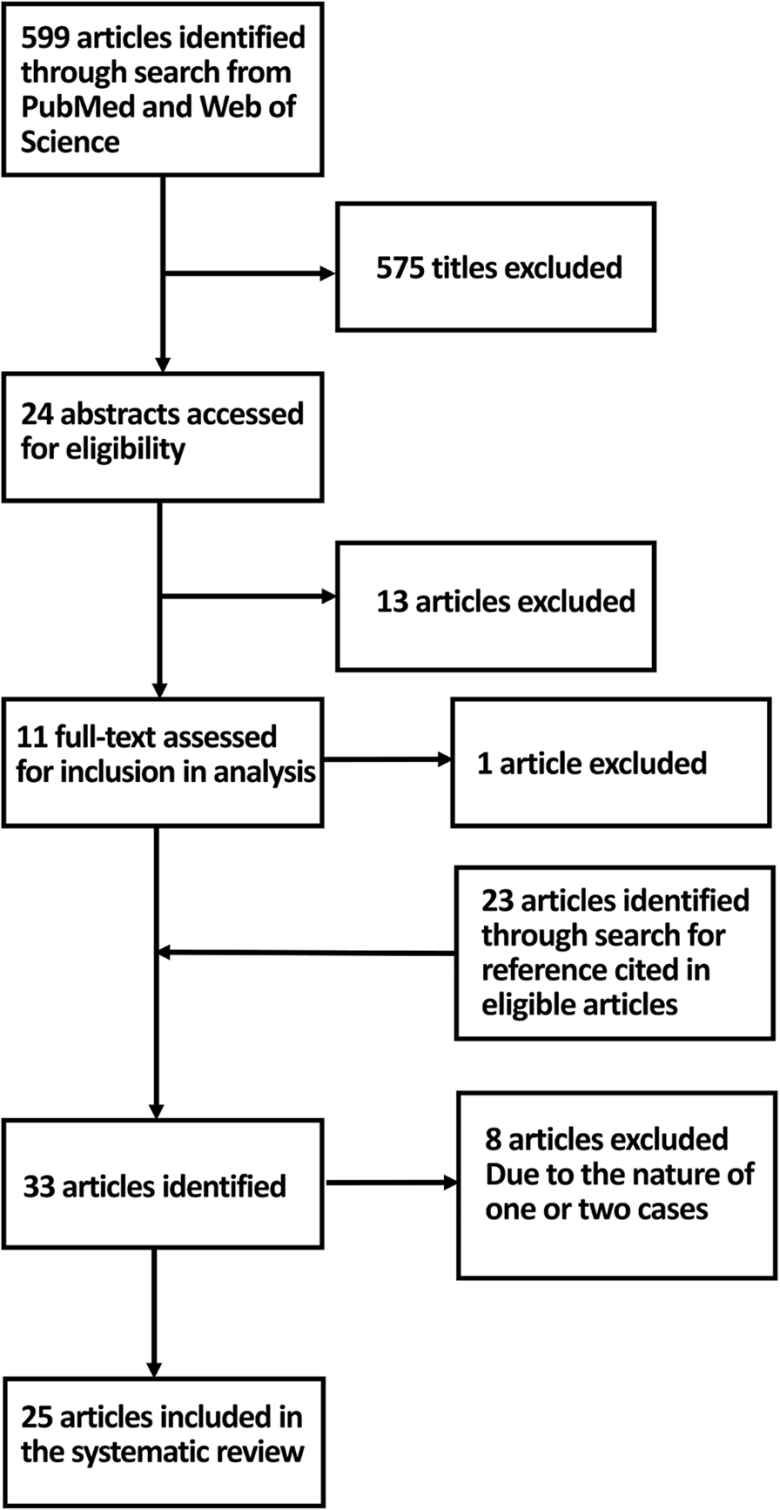
Flow diagram of study selection

The flow diagram of the search and study selection process is shown in Fig. 1. Among a total of 599 potentially relevant articles, 575 and 13 articles were excluded by screening of the titles and abstracts, respectively. One article was excluded by full-text screening. Following the same process for 23 additional manually identified articles, a total of 25 articles were selected as eligible articles [8, 14–37] and all were subject to meta-analysis. Of these, four studies were classified as case reports, four as reports of ICU cases, four as hospital outbreak studies, eight as retrospective studies and five as surveillance study. The majority of included articles were reported either from the KSA or the ROK, except for one study conducted in Jordan [22] and the WHO MERS-CoV Research Group that combined reports from multiple countries.

**Fig. 2:**
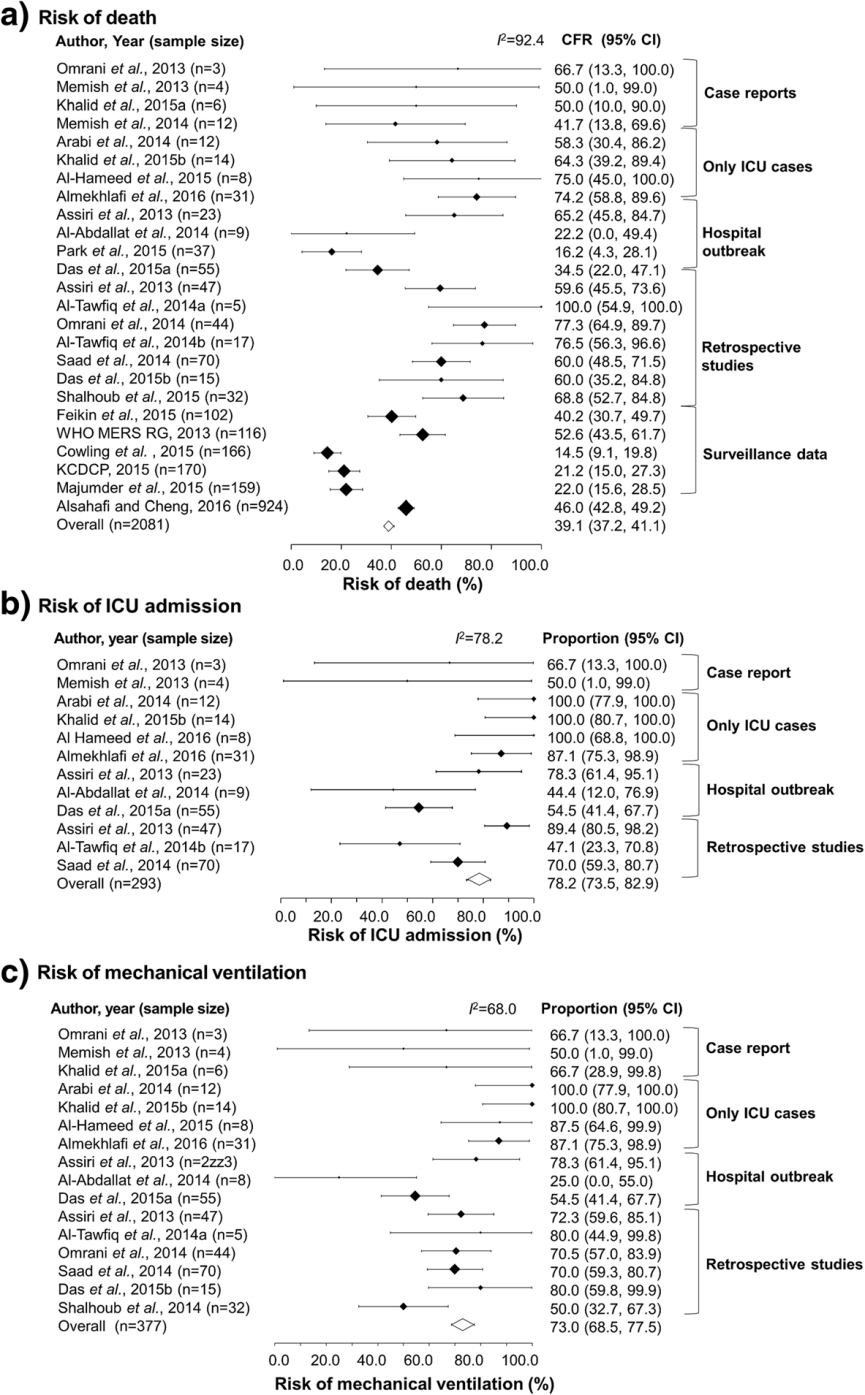
Estimated risks associated with Middle East respiratory syndrome (MERS) by published study. Panels show the risk estimates by study outcome: (**a**) risk of death, (**b**) risk of admission to the intensive care unit (ICU) and (**c**) risk of requiring mechanical ventilation. CFR represents the case fatality risk. The size of the diamonds reflects the sample size, and the whiskers extend to the lower and upper values of the 95% confidence interval (CI). The diamond without fill represents the pooled estimate using the inverse variance of the risk of death. I^2^ measures the extent of the heterogeneity, representing the proportion of variance in a meta-analysis that is attributable to study heterogeneity. Khalid et al., 2015a is [16] while Khalid et al., 2015b is [19]

The estimated CFR was reported in 25 articles, ranging from 14.5 to 100% (Fig. 2). The pooled CFR was 39.1% (95% CI: 37.2, 41.1), but the I2 was as large as 92.4%. The sample size of case reports ranged from 3 to 12, while studies with other designs tended to have larger samples, with 10 or more cases, except for one ICU study, one retrospective study and one hospital outbreak study. The proportions of ICU admission and mechanical ventilation among all cases were available in 12 and 16 articles, respectively. The proportion of ICU admission ranged from 44.4 to 100% with the pooled estimate at 78.2% (95% CI: 73.5, 82.9) and an I^2^ value of 78.2%. The proportion of mechanical ventilation ranged from 25.0 to 100% with the pooled estimate at 73.0% (95% CI: 68.5, 77.5) and an I^2^ value of 68.0%.

**Fig. 3:**
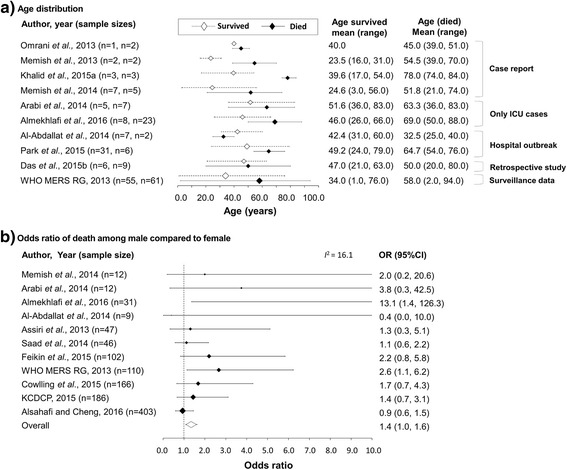
Age and sex distributions related to MERS-associated death by published study. **a** Ages of patients that survived and died of Middle East respiratory syndrome (MERS) are compared. The range represents the minimum and maximum age. **b** Odds ratio (OR) of MERS death among men compared with women. The size of the diamonds reflects the sample size, and the whiskers extend to the lower and upper values of the 95% confidence interval (CI). The vertical dashed line shows the threshold value of OR = 1. The diamond without fill represents the pooled estimate using the inverse variance of OR. Khalid et al., 2015a is [16]. ICU represents intensive care unit

Age and sex distributions are shown in relation to the risk of death by MERS in Fig. 3. In the majority of the studies (except for a study from Jordan), survivors were younger than those who died of MERS. Although not generally, infected men tended to die more often than women, and the pooled OR of death among men compared with women was 1.4 (95% CI: 1.1, 1.6). The I^2^ value of the sex difference for the risk of death was 48.6%.

**Fig. 4:**
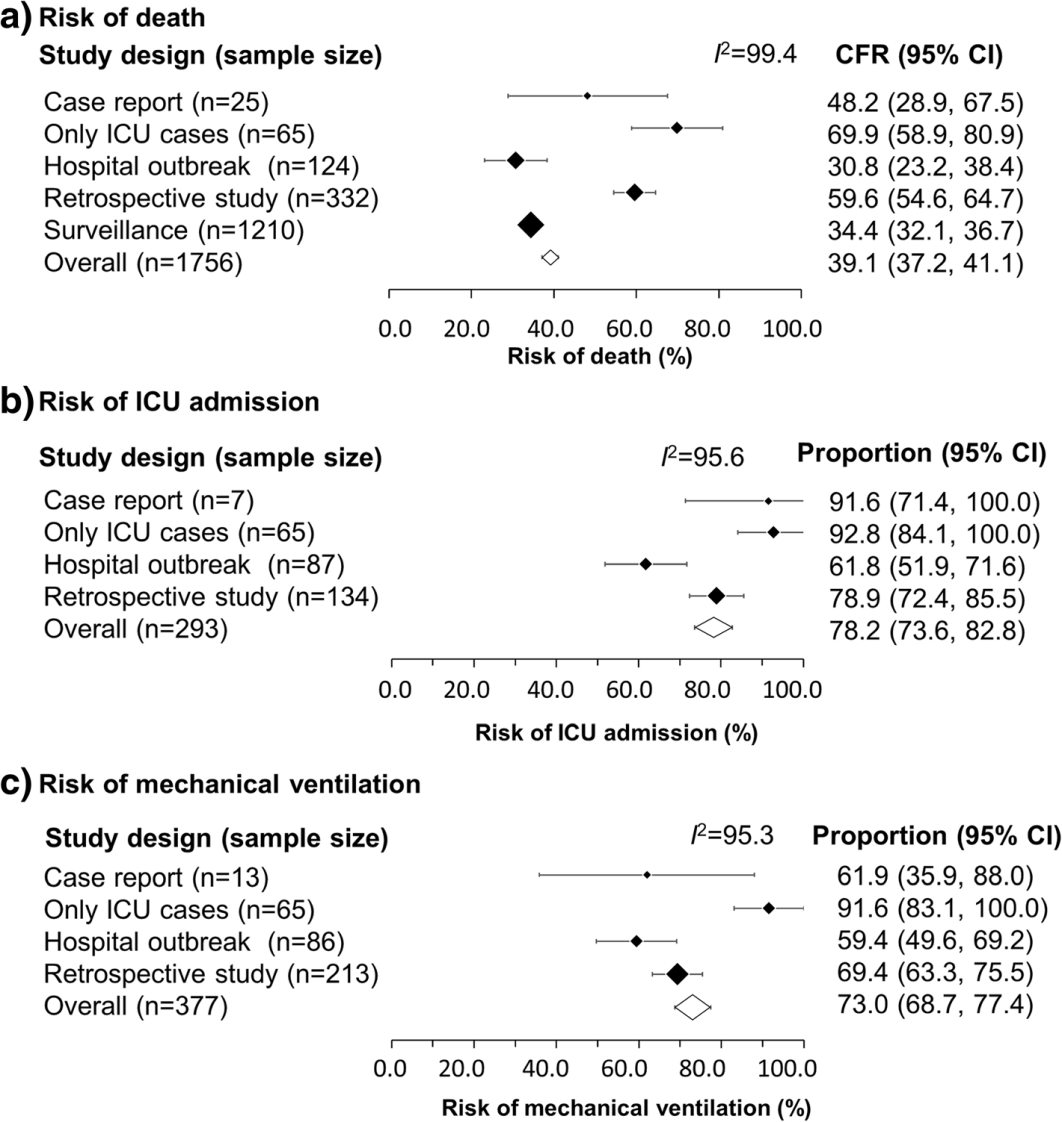
Estimated risks associated with Middle East respiratory syndrome (MERS) by study design. Panels show the risk estimates by study outcome: (**a**) risk of death, (**b**) risk of admission to intensive care unit (ICU) and (**c**) risk of mechanical ventilation. CFR represents the case fatality risk. The estimate for each study design represents the pooled risk of death calculated using the inverse variance of the risk of death in each published study. The size of the diamonds reflects the sample size, and the whiskers extend to the lower and upper values of the 95% confidence interval (CI). The diamond without fill represents the pooled estimate using the inverse variance of the risk of death. I^2^ measures the extent of the heterogeneity, representing the proportion of variance in a meta-analysis that is attributable to study heterogeneity

The risks of death, ICU admission and mechanical ventilation were stratified by study design and are shown in Fig. 4, in which the pooled estimate for each study design was compared. The risk of death in the hospital outbreak and surveillance studies was significantly smaller than in ICU case and retrospective studies. Risks of ICU admission and mechanical ventilation were the highest among ICU case studies, followed by case report and retrospective studies. Hospital outbreak studies yielded the smallest pooled risks of ICU admission and mechanical ventilation. When comparing surveillance-based data between KSA and ROK (Fig. 2), the risk of death in ROK (i.e., 14.5–22.0% [8, 33, 34]) tended to be lower than that in KSA (i.e., 46.0% by Alsahafi and Cheng [35]), perhaps reflecting the presence of the contact tracing effort in the ROK.

**Fig. 5:**
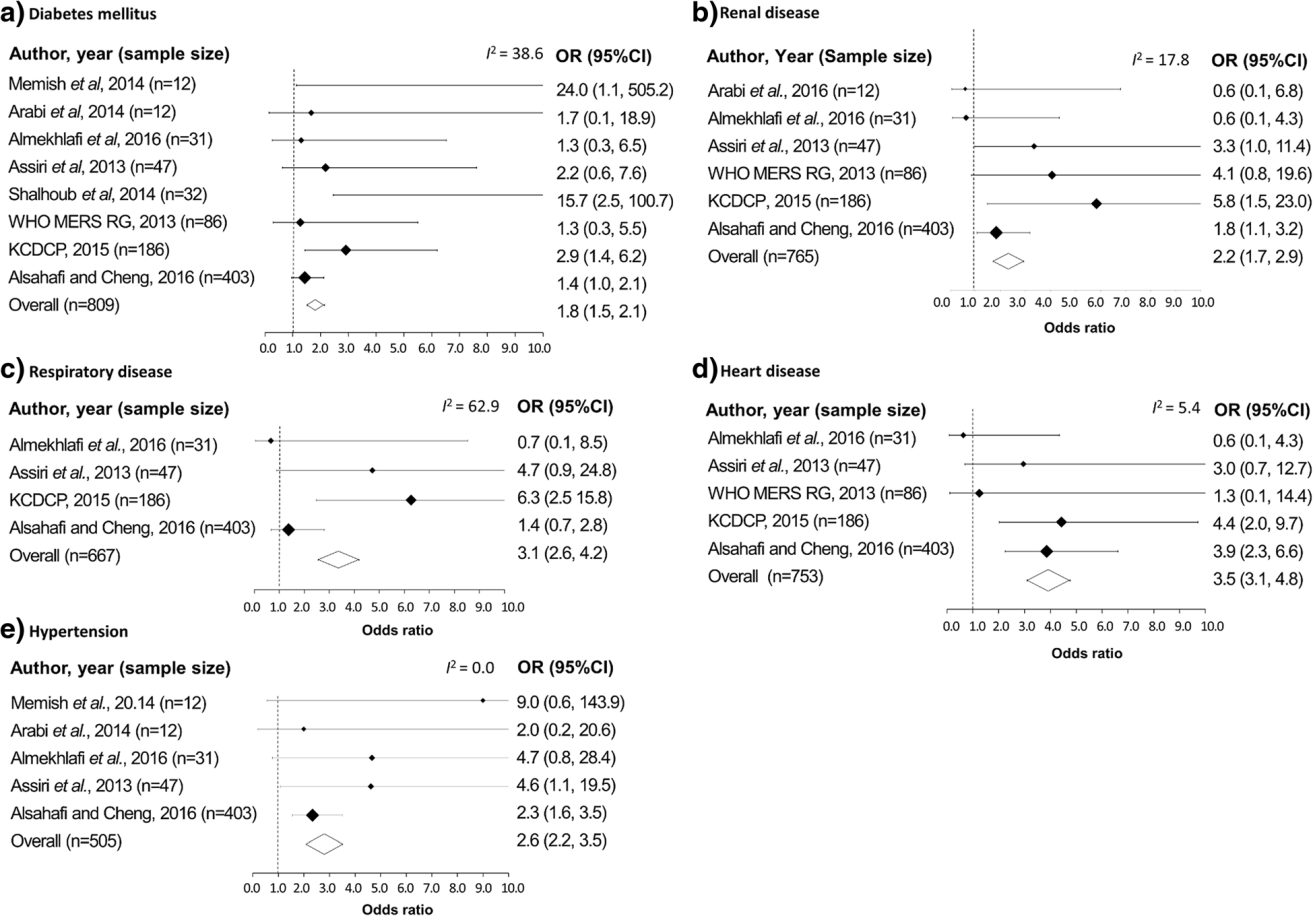
Risk of death by Middle East respiratory syndrome (MERS) by underlying medical condition. Panels **a**, **b**, **c**, **d**, and **e** show the risk estimates by underlying medical condition. The odds ratio (OR) for MERS death to compare those with underlying conditions against those without underlying conditions was calculated. The size of the diamonds reflects the sample size, and the whiskers extend to the lower and upper values of the 95% confidence interval (CI). The vertical dashed line shows the threshold value of OR = 1. The diamond without fill represents the pooled estimate using the inverse variance of the OR. I^2^ measures the extent of heterogeneity, representing the proportion of variance in a meta-analysis that is attributable to study heterogeneity

Figure 5 shows the possible association between five selected underlying medical conditions and the risk of death by MERS. Pooled estimates of the OR were greater than the value of 1 for all five comorbidities, including diabetes mellitus (*n* = 8 studies), renal disease (6 studies), respiratory disease (5 studies), heart disease (5 studies) and hypertension (5 studies). Among a total of five predictors, heart disease yielded the greatest OR value at 3.5 (95% CI: 3.1, 4.8) followed by respiratory disease with an OR of 3.1 (95% CI: 2.6, 4.2).

**Fig. 6:**
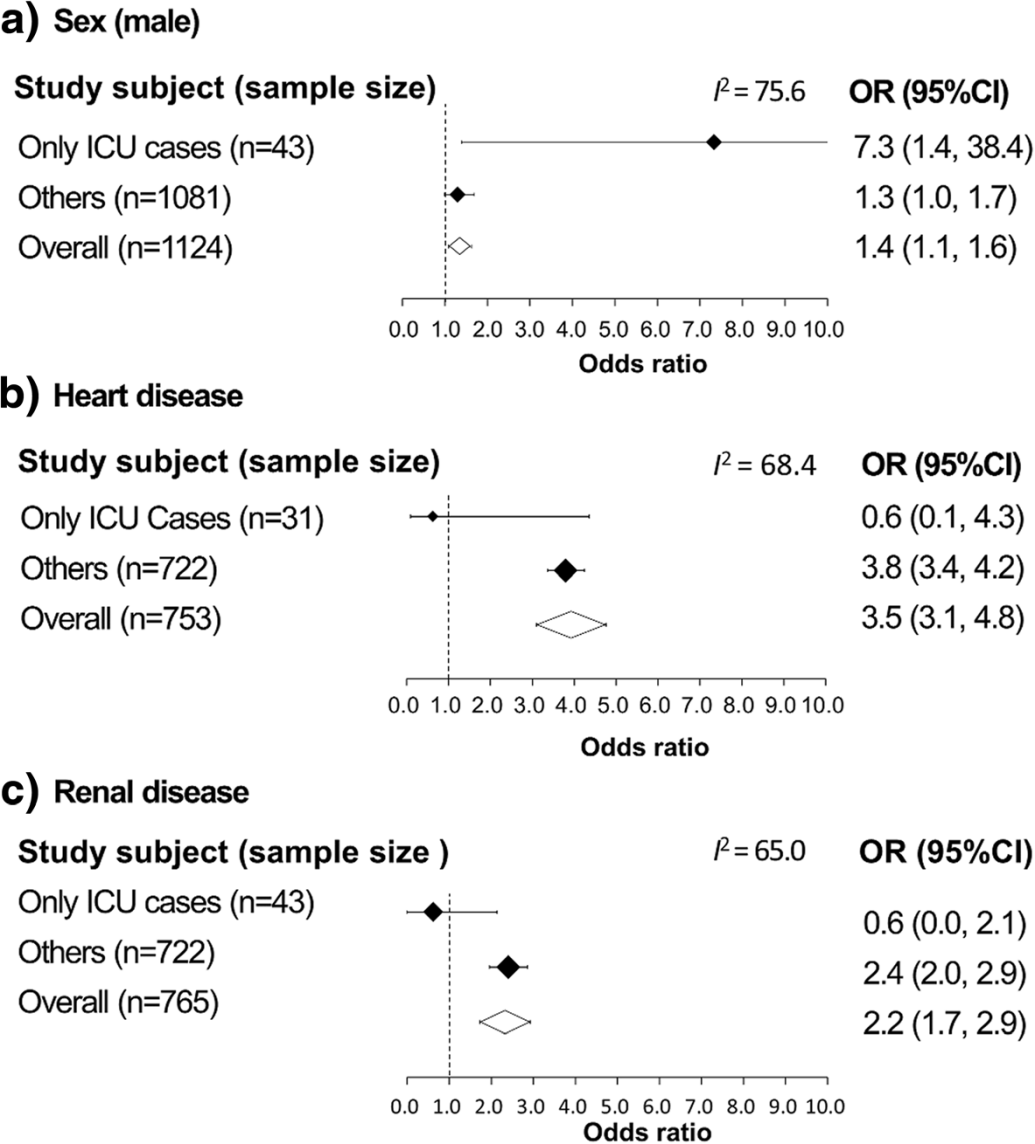
Risk factors of death by Middle East respiratory syndrome (MERS) by study design. Panels show the risk estimates by covariate: (**a**) sex, (**b**): heart disease and (**c**): renal disease). Odds ratio (OR) represents the odds ratio of death among men with underlying medical condition compared with women without comorbidities, respectively. The size of the diamonds reflects the sample size, and the whiskers extend to the lower and upper values of the 95% confidence interval (CI). The diamond without fill represents the pooled estimate using the inverse variance of the risk of death. I^2^ measures the extent of heterogeneity, representing the proportion of variance in a meta-analysis that is attributable to study heterogeneity

Figure 6 shows the potential association between the risk of death by MERS and potential predictors, including sex, heart disease and renal disease. Men from ICU studies tended to yield a greater OR for death compared with other study designs. Conversely, expected values of ORs for death among those with heart disease and renal disease compared with those without appeared to be lower than the value of 1.0.

## Discussion

The present study systematically reviewed the risk of severe manifestations and death by MERS by systematically searching and analyzing published articles from the KSA and the ROK and calculating not only the CFR but also the risks of ICU admission and requiring mechanical ventilation. Several clinical predictors of death were identified including older age, male sex and underlying medical conditions, including diabetes mellitus, renal disease, respiratory disease, heart disease and hypertension. The risk estimate appeared to vary by study design. In particular, studies focusing on patients in the ICU yielded the greatest estimates, while the CFRs for surveillance and hospital outbreak studies were smaller. These findings indicate that ascertainment biases in surveillance and hospital outbreak studies, frequently involving case finding effort, were smaller than in other types of studies. The importance of case finding effort is likely reflected in the different CFR estimates based on surveillance data between KSA and ROK. Although the presently identified clinical predictors are in line with previously published studies [6, 7, 11], the present study is the first to systematically analyze published studies, including clinical research studies, and extract findings that echo those of published articles. As was observed in this study, systematic search and analysis of the transmission characteristics [38] and spatial spreading patterns of MERS [39] have been successful.

An important contribution of the present study is that we demonstrated that the risk of death or severe manifestations is highly heterogeneous for various reasons, including different study designs. It is recognized that MERS involves asymptomatic infection [9], and thus, studies must be clear as to how the risk is estimated, including the definition and diagnostic methods used to identify infected individuals. Depending on the study design, the clinical predictors of death also differed. For example, renal and heart diseases might not predict the risk of death in an ICU setting, but they may be critically important in other settings that involve milder cases. Not only studies in ICU settings, but also retrospective studies yielded relatively high risk estimates for severe manifestations and death. Our finding raises concerns regarding the retrospective analysis of confirmed cases in registered databases without referring to biases associated with case detection and diagnosis, which could yield a biased risk estimate of MERS severity. In fact, that could explain why the CFR of confirmed cases among registered cases in patients’ database has been as high as 40%, while the CFR of secondary cases in the presence of contact tracing has been estimated at about 20% [6–10].

The comorbidities identified in our study are in line with those already identified elsewhere [6, 37]. The identification of comorbidities is not only stressed based on previous and present findings [11], but it is critically important to understand the underlying pathophysiological mechanisms. High representation of men among deceased cases may reflect the interaction of factors related to sex-specific lifestyle (e.g., smoking habits in the Middle East). Older age might reflect the greater likelihood of having underlying medical conditions. Diabetes, renal and respiratory diseases could predispose patients to be immunologically vulnerable and heart disease could induce water retention (e.g., secondary aldosteronism), both exacerbating the systemic condition. Hypertension could have been confounded by some other explanatory factor (s), for example, obesity could have likely led to both hypertension and MERS death. Nevertheless, identified predictors are accompanied by reasonable biological explanations.

The present study is not free from limitations. The biggest concern is, given the absence of identifying information, the included articles most likely referred to the same cases multiple times, potentially overestimating the number of cases. In fact, the total number of diagnosed and reported cases of MERS as of June 2016 is approximately 1,768 cases, but our systematic review included as many as 2,081 cases. Thus, it is likely that multiple reports from ROK (e.g., Cowling et al. [8], KCDCP [33] and Majumder et al. [34]) reported on the same cases multiple times. Rather, we did not avoid any overlap of cases in datasets because that adjustment forced us to adjust the overlap among the cases from the KSA in a similar manner. For this reason, the pooled estimate would never represent the actual pooled outcome data because the same case was counted multiple times. If we remove Cowling et al. [8] and Majumder et al. [34] from our analysis and include KCDCP [33], which had the largest sample size, the pooled estimate of the CFR would be increased to 45.4% (95% CI: 43.2, 47.7). This is understandable owing to the diminished impact of the extensive contact tracing effort in the ROK. Despite these overlaps, we conducted this systematic review to demonstrate that ascertainment biases likely act as a key factor that characterizes differential mortality across countries. To avoid any overlap of cases and better identify risk factors of ICU admission and death, it is advised to set up a common case registration system across countries and allocate identity number for each individual case.

As the second technical limitation to remember, it should be noted that the access to individual data was not achieved, and thus, for instance the age-related analysis did not rest on individual age data, and similarly, we have had limitations in the precision of the majority of outcome evaluations. Third, clinical predictors of death have been classified only at organ level, and moreover, individual behavioral factors or habitat [40] have not been examined in relation to the risk of MERS death. Fourth, non-English language manuscripts have been missed, and they include at least a few publications in Korea and one from Jordan.

Despite these problems, we cannot help but consider that the present study successfully and systematically evaluated the risk of severe manifestations and death by MERS by collecting published information on clinical predictors of the risk of death. An important consideration is that the associated risk estimation and identification of risk factors of MERS call for particular care in terms of study design, especially in aiming to eliminate biases associated with detection and diagnosis.

## Conclusions

Heterogeneity in risks of death and severe manifestations secondary to MERS was substantial. Differential study design was one of underlying reasons for the large heterogeneity. Statistical estimation of the risk of MERS death and identification of risk factors must be conducted with particular careful attention paid to study design, especially accounting for biases associated with case detection and diagnosis.

## Additional file

Additional file 1: Systematic review protocol. Summary of systematic review protocol is enumerated as a table. (DOCX 14 kb)

## Abbreviations

CFR: Case fatality risk
CI: Confidence interval
ICU: Intensive care unit
KSA: Kingdom of Saudi Arabia
MERS: Middle East respiratory syndrome
MERS-CoV: Middle East respiratory syndrome associated coronavirus
ROK: Republic of Korea
WHO: World Health Organization
