# Supplementary material for: Clinical determinants of the severity of Middle East respiratory syndrome (MERS): a systematic review and meta-analysis
Source: BMC Public Health. 2016 Nov 29;16:1203. doi: 10.1186/s12889-016-3881-4 (PMC5129628; doi:10.1186/s12889-016-3881-4)
Supplement: Additional file 1: — Systematic review protocol. Summary of systematic review protocol is enumerated as a table. (DOCX 14 kb) [file 12889_2016_3881_MOESM1_ESM.docx]

**Additional file 1 – Systematic review protocol**

Clinical determinants of the severity of Middle East respiratory syndrome (MERS): A systematic review and meta-analysis

Ryota Matsuyama, Hiroshi Nishiura, Satoshi Kutsuna, Kayoko Hayakawa, Norio Ohmagari

| Item | Details |
| --- | --- |
| Subject database | PubMed/Medline  Web of Science |
| Hand search | Implemented  Subjects: References that were cited in all included articles  Manual screening of all citations |
| Grey literature | Not included |
| Screening method | Primary screening: Title (by Ryota Matsuyama and Hiroshi Nishiura)  Secondary screening: Abstract |
| Classification of study design | Retrospective observational study |
| Bias risk and other assessment items | Nil |
| Assessment of evidence | Inconsistency in ascertainment |
| Method of metaanalysis | Fixed effects model |
| Effect measurement | Odds ratio and proportion of death (i.e. case fatality risk and admission to intensive care unit) |
| Any associated statistical analysis | Effect measurement plus 95% confidence interval  Forest plot |
